# Los test genéticos en atención primaria

**DOI:** 10.1016/j.aprim.2023.102695

**Published:** 2023-06-27

**Authors:** Sergio Fernández Torres, Ismael Ejarque Doménech

**Affiliations:** a Centro de Salud de Alaquàs, Valencia, España; b Centro de Salud de Almácera, Valencia, España; c Consulta de Genética Clínica del Hospital Vithas Aguas Vivas de Alzira, Valencia, España

*Sr. Editor:*

La genética clínica es una especialidad sanitaria en los países de nuestro entorno, pero no existe en España. Trata de una de las áreas fundamentales de la biología moderna con importantes aplicaciones en la práctica médica. El principal objetivo de esta especialidad es el estudio de las personas con alteraciones en el genoma, que es el responsable de contener la información necesaria para el correcto funcionamiento de nuestro organismo. Errores en esta información pueden originar enfermedades banales como el síndrome de Gilbert o más graves como la fibrosis quística o el cáncer hereditario. Estos genes que son el «software» del ser humano, que se transmiten de padres a hijos, también codifican proteínas que metabolizan o transportan los fármacos que hacen a los pacientes más o menos susceptibles a sus efectos secundarios o toxicidades indeseables. De esto se encarga la farmacogenética.

Las especialidades sanitarias que solicitan más test genéticos son oncología, ginecología, neurología, oftalmología, medicina interna, hematología, nefrología, cardiología, cirugía cardiovascular, endocrinología, alergología y neumología. Curiosamente medicina de familia y pediatría de atención primaria (AP) son las especialidades sanitarias que menos test genéticos solicitan y son, con diferencia, las que tienen una mayor visión holística y familiar de los pacientes, lo que lleva a poder detectar variantes patogénicas de genes familiares con mayor facilidad^1^. Es de innegable importancia formalizar y consolidar las habilidades y competencias de los residentes y adjuntos de medicina de familia y pediatras en genética clínica y enfermedades raras.

La genética clínica está viviendo una explosión de conocimiento que está ayudando a muchos pacientes a prevenir y mejorar muchas de estas enfermedades. Es pues innegable también la necesidad urgente del reconocimiento de la especialidad de genética clínica en España para la adecuada asimilación de esta revolución.

En la Comunidad Valenciana, en concreto en el Departamento del Hospital General, disponemos de una red pionera de consultas de genética en AP, que trabajan estrechamente y están supervisadas por la Unidad de Genética del Hospital General de Valencia. El centro de salud de Alaquàs ha sido el primero en abrir una consulta de genética en AP en noviembre de 2022 con excelentes resultados. ¿Qué hacen ya en estas nuevas consultas de genética en atención primaria? pues han realizado test genéticos en el propio centro de salud de: hipercolesterolemia familiar, hemocromatosis, síndrome de Gilbert, fibrosis quística, hipoacusia hereditaria no sindrómica, Parkinson de inicio temprano, enfermedad de Alzheimer familiar, aneurisma aórtico, displasia ectodérmica, farmacogenética (polimorfismo tolerancia a estatinas: gen SLCO1B1), derivación y seguimiento de sospecha de agregaciones familiares de cáncer más frecuentes (mama, colon, próstata…), asesoramiento preconcepcional y ampliación del estudio genético familiar. ¿A dónde queremos llegar? A ser una herramienta útil, rigurosa y efectiva para la detección de la mayoría de alteraciones genéticas más prevalentes y de fácil manejo de la población del departamento del Hospital General.

Los pacientes muestran un gran interés por el diagnóstico genético desde AP. Es una rama de la medicina muy gratificante; por ejemplo, a mediados de marzo de 2023 diagnosticamos desde nuestra consulta de genética en AP a un paciente varón de 65 años de edad con sordera desde la infancia. Le explicamos el tipo de mutación, el patrón de herencia mitocondrial, que el uso de antibióticos aminoglucósidos están contraindicados y que es aconsejable la ampliación del estudio familiar para prevenir la enfermedad en familiares que puedan tener esta mutación.

Las nuevas consultas de genética en AP trabajan en sintonía con la unidad de genética hospitalaria^2^ y ayudan a la realización de más estudios genéticos, garantizando la accesibilidad equitativa y evitando problemas de salud importantes en la población.
